# The International Hahnemannian Association: Third Annual Meeting

**Published:** 1883-09

**Authors:** J. B. Gregg Custis

**Affiliations:** Secretary


### INTERNATIONAL HAHNEMANNIAN ASSOCIATION.

The Association held its third annual meeting at the International Hotel, Niagara Falls, June 19th, 20th, and 21st.

On the first day the President delivered his annual address, entitled, "The Present and Future of Homœopathy." By resolution, copies were printed to be distributed by the Publication Committee.

The Secretary and Treasurer made their reports, which gave evidence that the Association was ahead in business and financial affairs.

The by-laws were so amended that the Association could meet wherever the majority directed, without regard to the meetings of the American Institute. So that a two-thirds vote was necessary for the election of a member; and that foreign members could vote by proxy in the election of officers.

At the afternoon session the following new members were elected, viz.: A. Chargé, M. D., of France; A. Matteoli, M. D., of Italy; Henry von Musits, M. D., of New York; J. A. Compton, M. D., of Indiana; Paz. Alvarez, M. D., of Spain; T. K. White, M. D., of England; A. McNeil, M. D., of Indiana, and G. M. Pease, M. D., of California.

The Bureau of Materia Medica reported provings of Ipecacuanha, Ledum palustra, and of Ovi gallina pellicula (membrane of egg-shell), by Dr. Swan. The Association resolved to prove Ovi pellicula, Dr. Swan offering to furnish the remedy. The report was followed by a most interesting and instructive discussion.

On Wednesday the Bureau of Clinical Medicine reported papers by Drs. H. N. Guernsey, Ad. Lippe, Benj. Ehrman, E. W. Berridge, C. Carlton Smith, L. M. Kenyon, C. F. Nichols, E. A. Ballard, Edw. Bayard, Sam'l Swan, R. R. Gregg, and G. Pompili. The report was followed by an especially good discussion.

The Bureau of Obstetrics reported papers from Drs. Mahoney, Foote, Rushmore, and Hayes, followed by a discussion bringing out important characteristics of remedies.

A committee was appointed to edit a volume containing the history, constitution, proceedings, etc., of the Association since 1881, with a list of members. Drs. Custis, Cranch, and Mills were appointed as said committee.

The Association then elected the following officers for the ensuing year, viz.: President, Geo. F. Foote, M. D., of Stamford, Conn.; Vice-President, R. R. Gregg, M. D., of Buffalo, N. Y.; Secretary, J. B. Gregg Custis, M. D., of Washington, D. C.; Treasurer, Edw. Cranch, M. D., of Erie, Pa.; Corresponding Secretary, E. W. Berridge, M. D., of London, England; Board of Censors, Dr. C. Pearson, of Washington, D. C., Chairman, with Drs. J. F. Smith, Sam'l Swan, C. H. Lawton, and Benj. Ehrman.

On Thursday afternoon the Bureau of Surgery reported papers from Drs. Cranch, Rushmore, L. B. Wells, and Ostrom.

It was resolved that the next meeting be held at the same place as that of the American Institute, but at least three days before.

The following were appointed Chairmen of the various Bureaus for the ensuing year, viz.: Materia Medica, J. P. Mills, M. D., of Chicago; Clinical Medicine, J. M. Biegler, M. D., of Rochester, N. Y.; Obstetrics and Diseases of Women, J. R. Haynes, M. D., of Indiapolis, Ind.; Surgery, C. H. Lawton, M. D., Wilmington, Del.

Letters were read from Drs. Lippe, Baer, Mahoney, and Foote, and the Association adjourned sine die.

J. B. Gregg Custis, *Sec'y.*

Note.—A more instructive or interesting meeting could not have been wished for than this, and the success of the Association is now fully assured.

The papers were all by representative men, full of practical lessons of value to the members.

An Association that can call forth such papers, and give its members such instructive meetings, need have no fear of opposition, nor does it need to detract from whatever of good may be in sister organizations. It will be noticed that the next meeting will be held three days before that of the Institute, and the Executive Committee promise that no pains shall be spared to fully repay any members or non-members who come to Deer Park at that time.

J. B. G. C., *Sec'y.*

