# Erratum

**Published:** 1883-10

**Authors:** 


Erratum.—Dr. E. Rushmore, not Dr. Mills, is Chairman of Bureau of Materia Medica, I. H. A.

